# Editorial: Dietary transformations and health implications among migrant populations

**DOI:** 10.3389/fnut.2026.2002042

**Published:** 2026-10-12

**Authors:** Megan A. Carney, Abiodun T. Atoloye, Alyshia Gálvez

**Affiliations:** 1 University of Arizona, Tucson, AZ, United States; 2 Utah State University, Logan, UT, United States; 3 Lehman College, New York, NY, United States

**Keywords:** food insecurity, health disparities, migration, structural vulnerability, migrant health

Across diverse histories and geographies, human migration nearly always implies dietary transformations. These changes unfold at multiple scales, from the individual and household, to collectives and entire societies. Relatedly, understanding dietary transformations requires also attending to the broader contexts of human migration and mobility in which they transpire. This Research Topic aims to deepen the understanding of how food environments shape migration trajectories, and in turn, how migration impacts food practices and health outcomes.

In his famous essay, “Food and Diaspora,” the US anthropologist Sidney Mintz (1) argued that people move with foods *and* that foods move with people. He suggested that these dual mobilities were foundational for understanding the modern food system and how people across the world eat today.

Mintz's (1) ideas remain ever-important as we consider the profound injustices characterizing the contemporary relationship between food and migration. While many people migrate as part of a larger household project to improve their access to economic and educational opportunities, migration is associated with food insecurity and can disrupt foodways, heightening lifetime risk for non-communicable chronic diseases. In many contexts, migrants experience greater food insecurity and higher risk of undernutrition than native-born residents, and long work hours and housing insecurity can disrupt preferred food routines. For migrants of precarious status, even access to basic nutritional sufficiency can be challenging. The articles in this Research Topic provide ample evidence of these injustices and how they register within the interconnected realms of diet, sociality, and health. They point to the structural vulnerabilities of migrant and diasporic populations that shape processes of dietary acculturation and render disproportionate burdens of food insecurity and non-communicable disease. Yet they also complicate depictions of these populations as “passive recipients” of dominant food environments and ideologies. Contrary to popular assumptions, migrant and diasporic populations are often agents in transforming the food practices of the places they move to.

The articles comprise a range of migration experiences (i.e., South-South and South-North migration), forms of evidence, research methods (i.e., survey- and statistically-based, ethnographic or qualitative), and critical perspectives (i.e., critical race and diaspora studies, feminist, decolonial, and global South scholarship).

Varre et al. help to set the stage in their mini-review of current dietary acculturation research, providing a global perspective while also warning against any flattening of differences. Their findings, sourced from 30 studies, highlight the significant risk factors for chronic disease among migrant populations. One of the more ethnographically-grounded contributions in the Research Topic is a study by García-Macías and Bárcenas based in Los Angeles, California that proposes the concept of “anticipatory governance of fear”—a structuring logic that permeates the dietary and health possibilities for undocumented Mexican migrants as they seek to evade the threat of immigration enforcement. Also in Los Angeles, Soleri et al. describe the resilience and creativity of migrants from Oaxaca residing in California to create a transnational food system responsive to their community's needs and preferences. Findings from community-based collaborative research in the US-Mexico borderlands inform calls by Carney et al. to heed the effects of compounding resource insecurities—particularly water insecurity—in shaping food practices and health outcomes of migrant and diasporic populations, including within the global North. While several pieces focus on dynamics in Mexican diasporic communities in the US borderlands, we also have important South-facing perspectives, with Rocha-Jimenez et al., as well as Fodde describing how transnational migrants from other parts of South America and the Caribbean navigate social exclusion, financial insecurity and challenging housing and working conditions in Buenos Aires and Santiago. Furthermore, Flórez et al. in their study among Dominican adults in the United States highlight the importance of strengthening culturally relevant food systems and community support as part of broader efforts to promote mental health equity. Radtke et al. underscore the importance of incorporating cultural food knowledge and ethnomedicinal beliefs into Food is Medicine initiatives and other culturally responsive nutrition interventions for immigrant populations. Phalle et al. examine food acculturation challenges in emerging education hubs such as India, and their findings underscore the need for culturally responsive nutrition support to help international students maintain diet quality and long-term health.

Taken together, the articles advance critical perspectives on health disparities and how we must move beyond deficit models to center structural conditions—legal precarity, labor exploitation, exclusion, etc.—that produce inequitable outcomes among migrant populations (García-Macías and Bárcenas; Rocha-Jimenez et al.). These conditions are inseparable from the industrialization of the global food system, a racial capitalist enterprise oriented toward profit rather than nourishment, which exploits migrant labor while depriving workers of resources to nourish themselves (Soleri et al.). Consequences are embodied: metabolic conditions as diseases of colonization manifest alongside declines in psychosocial wellbeing (Flórez et al.). Yet food also holds social significance, indexing relational processes shaped by migration, labor, memory, and educational experiences, with women as its stewards (Fodde; Carney et al.; Phalle et al.). For those dispossessed from the land, revitalizing or maintaining ancestral food practices offers healing and therapeutic potential (Radtke et al.), as dietary transformations may index resistance and/or struggles for belonging (Fodde; García-Macías and Bárcenas). Across the board, authors in this Research Topic call for rethinking how scholars, practitioners, and policymakers frame and intervene in the food and health practices of migrant populations.
